# Effects of missense mutations in sortase A gene on enzyme activity in *Streptococcus mutans*

**DOI:** 10.1186/s12903-016-0204-1

**Published:** 2016-04-11

**Authors:** P. L. Zhuang, L. X. Yu, Y. Tao, Y. Zhou, Q. H. Zhi, H. C. Lin

**Affiliations:** Department of Preventive Dentistry, Guanghua School of Stomatology, Sun Yat-Sen University, 56 Ling Yuan Road West, Guangzhou, China; Guangdong Provincial Key Laboratory of Stomatology, Sun Yat-Sen University, Guangzhou, China; Department of Stomatology, Sun Yat-Sen Memorial Hospital, Sun Yat-Sen University, 107 Yan Jiang Road West, Guangzhou, China

**Keywords:** Caries, Missense mutation, *srtA*, *Streptococcus mutans*, Enzyme activity

## Abstract

**Background:**

*Streptococcus mutans* (*S. mutans*) is the major aetiological agent of dental caries, and the transpeptidase Sortase A (SrtA) plays a major role in cariogenicity. The T168G and G470A missense mutations in the *srtA* gene may be linked to caries susceptibility, as demonstrated in our previous studies. This study aimed to investigate the effects of these missense mutations of the *srtA* gene on SrtA enzyme activity in *S. mutans*.

**Methods:**

The point mutated recombinant *S.mutans* T168G and G470A sortases were expressed in expression plasmid pET32a. *S. mutans* UA159 sortase coding gene *srtA* was used as the template for point mutation. Enzymatic activity was assessed by quantifying increases in the fluorescence intensity generated when a substrate Dabcyl-QALPNTGEE-Edans was cleaved by SrtA. The kinetic constants were calculated based on the curve fit for the Michaelis-Menten equation.

**Results:**

SrtA_△N40(UA159)_ and the mutant enzymes, SrtA_△N40(D56E)_ and SrtA_△N40(R157H)_, were expressed and purified. A kinetic analysis showed that the affinity of SrtA_△N40(D56E)_ and SrtA_△N40(R157H)_ remained approximately equal to the affinity of SrtA_△N40(UA159)_, as determined by the Michaelis constant (*K*_*m*_). However, the catalytic rate constant (*k*_*cat*_) and catalytic efficiency (*k*_*cat*_*/K*_*m*_) of SrtA_△N40(D56E)_ were reduced compared with those of SrtA_△N40(R157H)_ and SrtA_△N40(UA159)_, whereas the *k*_*cat*_ and *k*_*cat*_*/K*_*m*_ values of SrtA_△N40(R157H)_ were slightly lower than those of SrtA_△N40(UA159)_.

**Conclusions:**

The findings of this study indicate that the T168G missense mutation of the *srtA* gene results in a significant reduction in enzymatic activity compared with *S. mutans* UA159, suggesting that the T168G missense mutation of the *srtA* gene may be related to low cariogenicity.

**Electronic supplementary material:**

The online version of this article (doi:10.1186/s12903-016-0204-1) contains supplementary material, which is available to authorized users.

## Background

Dental caries is an infective transmittable bacterial disease characterized by a multi-factorial pathology, and *Streptococcus mutans* (*S. mutans*) is considered as the primary aetiological agent of dental caries [1, 2]. Adhesion to a tooth surface and biofilm formation by *S. mutans* are the initial steps in caries development [2]. Pac (also called P1 and SpaP) is a multi-functional adhesive and is considered the primary factor that mediates the early attachment to tooth enamel [3]. Glucan binding protein C (GbpC), wall-associated protein A (wapA) and dextranase have been demonstrated to be closely related to adherence and biofilm properties [4–6]. The aforementioned proteins all contain a conserved LPXTG motif [7, 8]. The sortase A (SrtA) enzyme has been demonstrated as an essential transpeptidase that recognizes the LPXTG motif and responsible for sorting and anchoring those proteins to the cell wall of *S. mutans* [9]. Inactivation of the *srtA* gene could result in defective pathogenesis [10]. For example, Pac from *S. mutans srtA* inactivated strain could not attach to cell wall, which inhibits the ability of the mutant strain to colonize teeth and form a biofilm, and consequently reduces the occurrence of caries [11, 12]. Therefore, SrtA is thought to take a critical role in pathogenesis of *S. mutans*.

The various genotypes of *S. mutans* are involved in the susceptibility to dental decay [13, 14], and the distribution of genotypes of *S. mutans* differs by population. In our previous studies, we compared the *srtA* gene of *S. mutans* strains isolated from caries-free children and children with high-severity caries. Chromosomal DNA of *S. mutans* strains were extracted and amplified by PCR (polymerase chain reaction) to obtain the *srtA* gene. Then the purified PCR products were sequenced. The *srtA* gene sequence of *S. mutans* UA159 was selected as a reference sequence. The *srtA* gene sequences of *S. mutans* clinical isolates were compared with that of *S. mutans* UA159 using Variant Reporter™ Software (Applied Biosystems, CA, USA) (accession numbers: KP301259 - KP301500). The distributions of missense mutations were compared between the groups [15, 16]. A total of 17 missense mutation sites were found and remarkably, the prevalence of the point mutations T168G and G470A significantly differed between the two groups [16]. The total length of the *srtA* gene in *S. mutans* UA159 is 741 bp. T168G is a point mutation at the 168th base in the *srtA* gene; this base was T in *S. mutans* UA159, while some clinical isolates had a G base substitution at that site. Additionally, G470A denotes a G base at the 470th base in the *srtA* gene of *S. mutans* UA159, while an A base is substituted in the *srtA* gene of some clinical isolates. The frequency of mutations at the 168 locus was significantly higher in the caries-free group than in the high-severity caries group. Moreover, strains with the locus 470 polymorphism exhibited a significantly higher mutation frequency in the high-severity caries group.

Since SrtA is closely associated with adherence and biofilm formation, we hypothesized that the missense mutations T168G and G470A in the *srtA* gene might affect the function of the SrtA enzyme and consequently lead to the changes in the cariogenicity of *S.mutans*. Based on our previous study, we constructed T168G and G470A missense mutations using the *srtA* gene of *S. mutans* UA159 as a template, and investigated the effects of the two missense mutations on SrtA activity in *S. mutans*.

## Methods

### Bacterial strains, plasmids, and culture conditions

*S. mutans* UA159 (ATCC700610) (Guangdong Culture Collection Centre of Microbiology, Guangzhou, China) was used as the source of chromosomal DNA for the PCR. The *Escherichia coli* (*E. coli*) BL21 (TaKaRa, Kyoto, Japan) as a host of gene operation and expression vector pET32a (Novagen, Madison, WI, USA) were used for gene expression. *E. coli* BL21 strains were grown in Luria-Bertani (LB) broth and plated onto LB medium containing 1.5 % (w/v) agar at 37 °C. Ampicillin was added when needed at 100 μg/mL (final concentration).

### Construction of *srtA*_*△N120(UA159)*_ and mutant expression vectors

SrtA is a membrane-anchoring protein containing an N-terminal signal peptide that can decrease its hydrophilicity. Therefore, full-length SrtA is difficult to purify and is unstable [17]. However, the transpeptidase activity of the truncated SrtA enzyme is not influenced by the absence of the N-terminal signal peptide because the deleted hydrophobic N-terminal region of SrtA functions as a signal peptide for secretion and a stop-transfer signal for membrane anchoring [18, 19]. Thus, to decrease the hydrophobicity of SrtA, the truncated SrtA lacking the N-terminal 40 amino acids was expressed in this study according to previous studies [20–22].

This study protocol was approved by the Ethics Committee of Guanghua School of Stomatology, Sun Yat-sen University (ERC-[2012]-13). Based on our previous epidemiological investigation [16], *srtA*_*△N120(T168G)*_ and *srtA*_*△N120 (G470A)*_ were constructed using the *srtA* gene of *S. mutans* UA159 as a template. The chromosomal DNA of *S. mutans* UA159 was extracted and amplified DNA fragment which contains truncated SrtA coding gene according to previously described methods [20, 21] with modifications. In brief, the primers 5′-CG*GGATCC* GCTTGGAATACCAATAGATATCAG-3′ (*BamH*I site is italic) and 5′-CCG*CTCGAG* TTAAAATGATATTTGATTATAGGACTGC-3′ (*Xho*I site is italic) were used to amplify the truncated *srtA* fragment (621 bp) from *S. mutans* UA159 chromosomal DNA by PCR. The *srtA* fragment was cloned into linearized pET32a vector by digested with *BamH*I and *Xho*I to generate the X6 HIS tagged recombinant plasmid pET32a-*srtA*_*△N120(UA159)*_. The site-directed mutagenesis of T168G and G470A was performed using the MutanBEST Kit (TaKaRa, Kyoto, Japan) with pET32a-*srtA*_*△N120(UA159)*_ as a template plasmid to construct pET32a-*srtA*_*△N120(T168G)*_ and pET32a-*srtA*_*△N120(G470A)*_ by following the manufacturer’s instructions. The primers 5′-GCAAGAAAGAG*G* ATTGAACACAACAAGGC-3′ (mutated base is italic) and 5′-TAACATTAGAAACCTG ATATCTATTGGTATTCCAAG-3′ were used to generate the T168G mutation, and the primers 5′-CCTTTAGAAC*A*TGCAAAAGAAGGCATGG-3′ (mutated base is italic) and 5′-TGAAAAGAGCATCTGTGAAGATCCGGTC-3′ were used to generate the G470A mutation. As expressed products of these gene mutations, the D56E and R157H mutants of SrtA were generated. All primers used in this study were synthesised by Shanghai Sangon Company (Shanghai, China). The plasmids were sequenced by Shanghai Sangon Company to verify that the expected sites were mutated.

### Expression and purification of SrtA_△N40(UA159)_ and mutant enzymes

The pET32a-*srtA*_*△N120(UA159)*_ and mutant constructs were introduced into *E. coli* BL21 by chemical transformation by the manufacturer’s protocol. The transformed cells were grown in Luria broth medium containing 50 μg/mL ampicillin at 37 °C until the OD_600_ reached 0.6. The expression of truncated SrtA in *E. coli* BL21 was induced with 0.2 mM isopropyl β-D-1-thiogalactopyranoside (IPTG), and the cells were grown for another 6 h at 15 °C. The supernatant was then collected and centrifuged at 16,000 × g for 15 min. All soluble recombinant SrtA enzymes were purified on a Ni Sepharose 6 Fast Flow column (GE healthcare, Piscataway, NJ, USA) according to the manufacturer’s instructions. The purity and specificity of the SrtA_△N40(UA159)_ and mutant enzyme preparations were verified by sodium dodecyl sulphate–polyacrylamide gel electrophoresis (SDS-PAGE) and Western blot analysis using a rabbit anti-His tag monoclonal antibody (Abgent, San Diego, CA, USA).

### Non-denaturing polyacrylamide Gel analysis

The soluble recombinant SrtA enzymes were subjected to non-denaturing polyacrylamide gel electrophoresis (native PAGE) described previously [23, 24] with modifications. The proteins were loaded onto 4–16 % Bis-Tris gels and resolved by electrophoresis at 4 °C. The gels were stained with Coomassie Brilliant Blue R250 (Bio-Rad, Hercules, CA, USA) and the protein bands were visualized. The ratios of dimers/monomers of SrtA proteins were evaluated by comparing the densities of dimer bands and monomer bands using ImageJ software (National Institutes of Health, Bethesda, MD, USA). All the reported ratios are the means of triplicate assays.

### SrtA activity assay

The activities of the purified SrtA_△N40(UA159)_ and the two point mutated enzymes were monitored as described previously [21, 22] with modifications. The synthetic peptide 4-(4-dimethylamino phenylazo) benzoic acid (Dabcyl)-QALPETGEE-5-[(2-aminoethyl)amino]naphthalene-1-sulphonic acid (Edans) (Dabcyl-QALPNTGEE-Edans) (Jiershenghua, Shanghai, China) was used as the substrate to determine SrtA activity. The substrate Dabcyl-QALPNTGEE-Edans contains a fluorescent luminophore and a fluorescence quencher. When Dabcyl-QALPNTGEE-Edans is cleaved by SrtA, the fluorophore Edans group is separated from the quencher Dabcyl group, which enhances the fluorescence signal. Dabcyl-QALPNTGEE-Edans was added to the kinetic reaction at a final concentration from 0.2 μM to 12.8 μM. Reactions were performed in 400 μL of reaction buffer (50 mM Tris–HCl, 5 mM CaCl_2_, 150 mM NaCl, pH 7.5) containing varying concentrations of fluorescent peptide substrate Dabcyl-QALPNTGEE-Edans (0.2-12.8 μM), 1.2 μM purified SrtA, and 0.2 M NH_2_OH. The experiments were performed for 30 min at 37 °C at an excitation wavelength at 350 nm and an emission wavelength at 495 nm. SrtA activity was assessed by quantifying increases in fluorescence intensity using a Victor^3^ 1420 multilabel counter (PerkinElmer, Waltham, MA, USA). The maximum velocity (*V*_*max*_) and Michaelis constant (*K*_*m*_) were calculated based on the curve fit for the Michaelis-Menten equation using Origin 8 software (OriginLab, Northampton, MA, USA):

$$ v= Vmax\left[S\right]/\left({K}_m+\left[S\right]\right) $$

where *v* is the slope during the linear phase of cleavage and *[S]* is the substrate concentration. The catalytic rate constant (*k*_*cat*_) was calculated based on the ratio of *V*_*max*_ to the enzyme concentration, and the catalytic efficiency was determined based on the *k*_*cat*_*/K*_*m*_ ratio. All reported values are the means of triplicate assays.

## Results

### Site-specific mutation of *srtA*_*△N120(UA159)*_

**Fig. 1:**
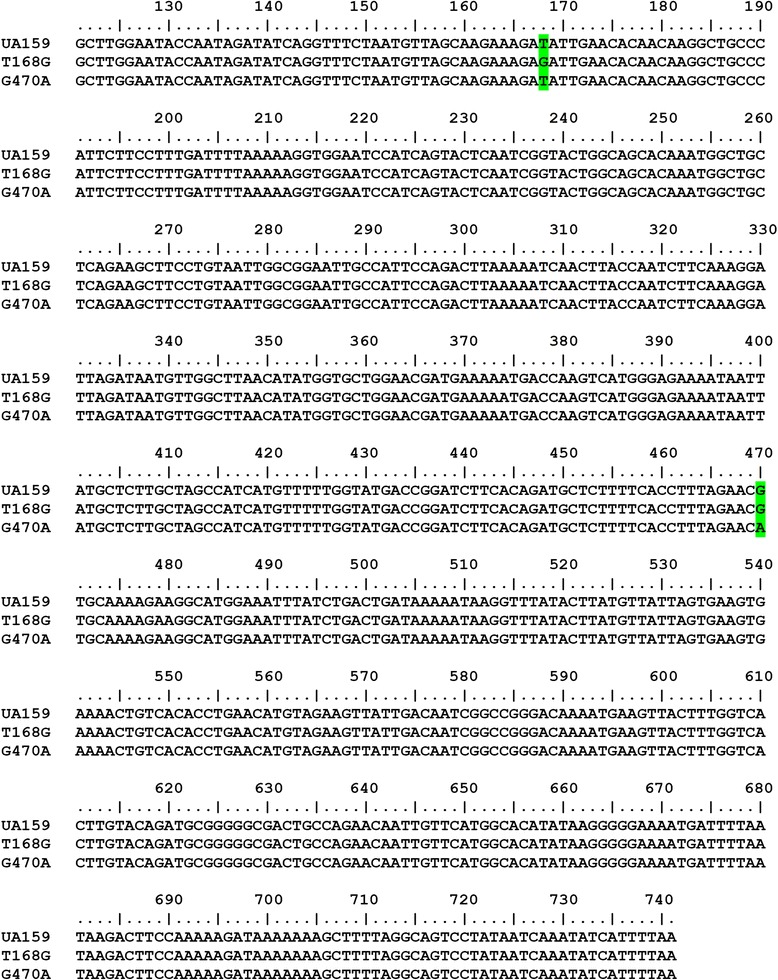
Point mutations in mutants compared with the *srtA* gene of *S. mutans* UA159. Detailed legend: *srtA*_*△N120(T168G)*_ contains a point mutation at base 168, and *srtA*_*△N120 (G470A)*_ contains a point mutation at base 470 (labelled in green)

The sortase coding gene *srtA*_*△N120(T168G)*_ and *srtA*_*△N120(G470A)*_ were generated by point mutation procedure from *srtA* gene of *S. mutans* UA159. The mutated nucleotide positions in each *srtA* genes were indicated in Fig. 1.

### Expression and purification of SrtA_△N40 (UA159)_ and mutants

**Fig. 2:**
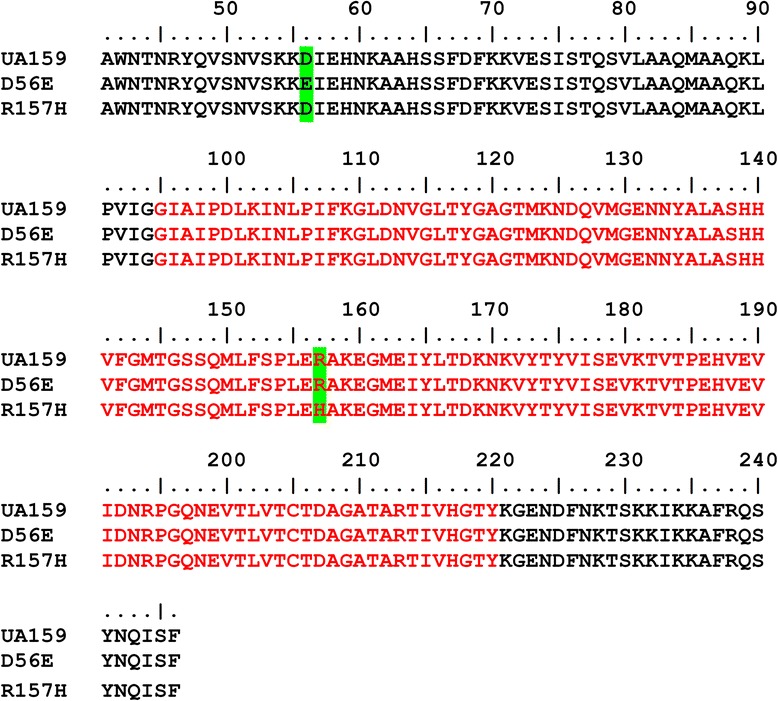
Point mutations in mutant enzymes compared with the SrtA enzyme of *S. mutans* UA159. Detailed legend: SrtA_△N40(D56E)_ contains a single mutation at the 56th amino acid residue, and SrtA_△N40(R157H)_ contains a single mutation at the 157th amino acid residue (labelled in green). The putative catalytic domain of SrtA is shown in red

**Fig. 3:**
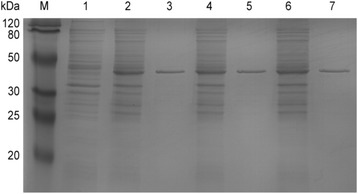
SDS-PAGE analysis of SrtA enzyme expression. Detailed legend: Expression of non-purified and purified SrtA analysed by SDS-PAGE. Lane M: Protein marker. Lane 1: Supernatant from *E. coli* BL21 transfected with pET32a. Lanes 2, 4, 6: Supernatants from *E. coli* BL21 transfected with pET32a-*srtA*_*△N120(UA159)*_, pET32a-*srtA*_*△N120(T168G*)_ and pET32a-*srtA*_*△N120(G470A)*_, respectively, before purification. Lanes 3, 5, 7: Supernatants from *E. coli* BL21 transfected with pET32a-*srtA*_*△N120(UA159)*_, pET32a-*srtA*_*△N120(T168G)*_ and pET32a-*srtA*_*△N120(G470A)*_, respectively, after purification using Ni sepharose 6 Fast Flow columns. New bands appeared in the supernatants from *E. coli* BL21 containing pET32a-*srtA*_*△N120(UA159)*_, pET32a-*srtA*_*△N120(T168G)*_ and pET32a-*srtA*_*△N120(G470A)*_ compared with the supernatant from *E. coli* BL21 containing pET32a. The estimated molecular weight of SrtA_△N40(UA159)_ and the mutant enzymes was approximately 42 kDa as indicated by SDS-PAGE

The SrtA_△N40(UA159)_ and point mutated sortase SrtA_△N40(D56E)_ and SrtA_△N40(R157H)_ were expressed as the recombinant protein that coded by *srtA*_*△N120(UA159)*_, *srtA*_*△N120(T168G)*_ and *srtA*_*△N120 (G470A)*_, respectively. Compared with the amino acid sequence of SrtA_△N40(UA159)_, the mutant enzyme SrtA_△N40(D56E)_ contains a single amino acid substitution from aspartate (D) to glutamate (E) at the 56th amino acid residue, while SrtA_△N40(R157H)_ contains a single amino acid substitution from arginine (R) to histidine (H) at the 157th amino acid residue. The amino acid sequences are shown in Fig. 2. SDS-PAGE analysis of expressed and purified SrtA_△N40(UA159),_ SrtA_△N40(D56E)_ and SrtA_△N40(R157H)_ is shown in Fig. 3.

**Fig. 4:**
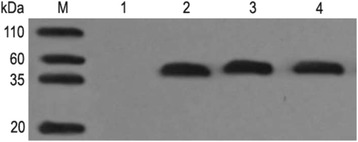
Western blot of SrtA_△N40(UA159),_ SrtA_△N40(D56E)_ and SrtA_△N40(R157H)_ probed with rabbit anti-His tag monoclonal antibody. Detailed legend: Lane M: Marker. Lane 1: Supernatant from *E. coli* BL21 transfected with pET32a. Lanes 2, 3, 4: Supernatants from *E. coli* BL21 transfected with pET32a-*srtA*_*△N120(UA159)*_, pET32a-*srtA*_*△N120(T168G)*_ and pET32a-*srtA*_*△N120(G470A)*_, respectively

As shown in Fig. 3, the estimated molecular weight of the purified enzymes was approximately 42 kDa, which was consistent with the theoretical molecular weight. Western blot analysis for SrtA_△N40(UA159),_ SrtA_△N40(D56E)_ and SrtA_△N40(R157H)_ probed with a rabbit anti-His tag monoclonal antibody revealed anti-His antibody-reactive bands (Fig. 4).

### Native PAGE analysis

**Fig. 5:**
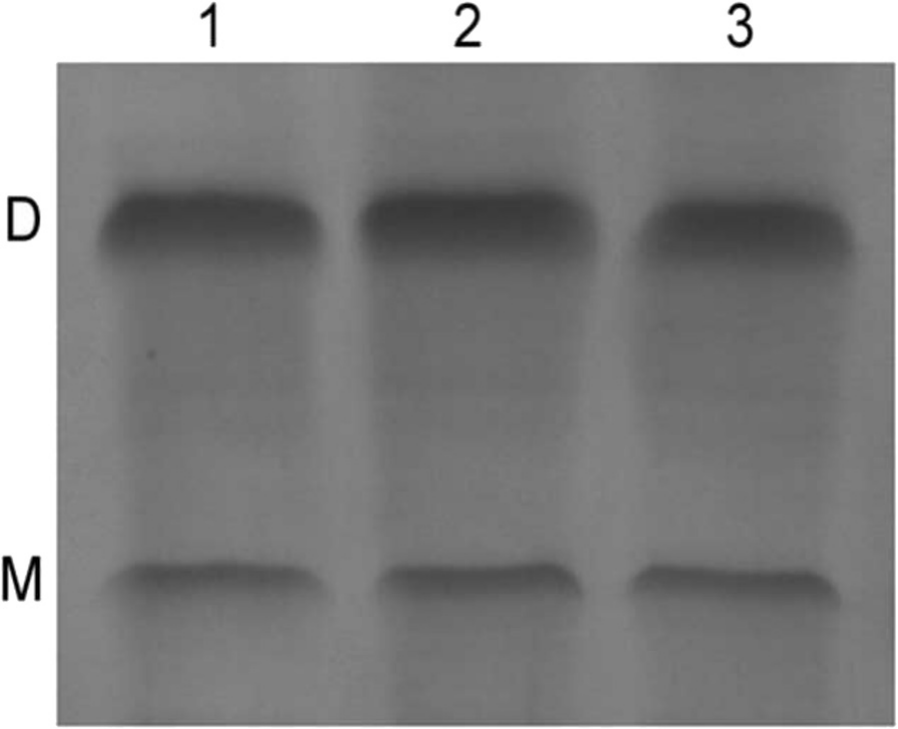
Native PAGE of SrtA_△N40(UA159)_ and mutant proteins. Detailed legend: Lane 1: Dimer and monomer of SrtA_△N40(UA159)_. Lane 2: Dimer and monomer of SrtA_△N40(D56E)_. Lane 3: Dimer and monomer of SrtA_△N40(R157H)_. The band labelled with D indicates the band of dimeric protein; the band labelled with M indicates the band of monomeric protein

Native PAGE was used to evaluate the native status of purified SrtA_△N40(UA159)_ and mutants. The dimer/monomer ratios of recombinant SrtA enzymes were estimated by comparing the densities of dimer bands and monomer bands. As shown in Fig. 5, the recombinant SrtA enzymes primarily existed as monomers and dimers. The dimer/monomer ratios of SrtA_△N40(UA159)_, SrtA_△N40(D56E)_ and SrtA_△N40(R157H)_ were 3.25 ± 0.16, 3.28 ± 0.25 and 3.21 ± 0.32, respectively. The dimer/monomer ratios of SrtA_△N40(D56E)_ and SrtA_△N40(R157H)_ were close to that of SrtA_△N40(UA159)_ on native gel.

### SrtA activity assay

To measure the rate of cleavage by SrtA_△N40(UA159),_ SrtA_△N40(D56E)_ and SrtA_△N40(R157H)_, the substrate Dabcyl-QALPNTGEE-Edans was incubated with the purified enzymes, and the kinetic constants were calculated for the hydrolysis catalysed by SrtA_△N40(UA159)_ and the mutant enzymes.

The kinetic parameters for SrtA_△N40(UA159),_ SrtA_△N40(D56E)_ and SrtA_△N40(R157H)_ are compared in Table 1. The cleavage activity of the mutant enzymes was reduced compared with SrtA_△N40(UA159)_, and the cleavage activity of SrtA_△N40(R157H)_ was more similar to SrtA_△N40(UA159)_ than SrtA_△N40(D56E)_.

**Table 1:** Comparison of the enzymatic activities of SrtA_△N40(UA159)_ and the mutant enzymes

Enzyme	*V*_*max*_ (μM · s^-1^) (×10^-3^)	*k*_*cat*_ (s^-1^) (×10^-3^)	*K*_*m*_ (μM)	*k*_*cat*_ */K*_*m*_ (μM^-1^ · s^-1^) (×10^-4^)
SrtA_△N40(UA159)_	3.75 ± 0.15	3.04 ± 0.12	15.50 ± 0.25	1.96 ± 0.08
SrtA_△N40(D56E)_	0.91 ± 0.13	0.74 ± 0. 11	15.34 ± 0.32	0.48 ± 0.06
SrtA_△N40(R157H)_	2.78 ± 0.26	2.25 ± 0.21	15.35 ± 0.07	1.47 ± 0.14

The *k*_*cat*_ values of SrtA_△N40(UA159)_ and SrtA_△N40(R157H)_ were approximately 3.1-fold and 2.0-fold higher than the *k*_*cat*_ of SrtA_△N40(D56E)_, respectively, whereas the *k*_*cat*_ of SrtA_△N40(UA159)_ was only 0.3-fold higher than the *k*_*cat*_ of SrtA_△N40(R157H)_.

The *K*_*m*_ values of SrtA_△N40(D56E)_ and SrtA_△N40(R157H)_ showed negligible decreases compared with the *K*_*m*_ of SrtA_△N40(UA159),_ suggesting that the affinities of SrtA_△N40(D56E)_ and SrtA_△N40(R157H)_ for substrate Dabcyl-QALPNTGEE-Edans were approximately equal to those of SrtA_△N40(UA159)_.

SrtA_△N40(UA159)_ and SrtA_△N40(R157H)_ catalysed the sorting reaction more efficiently than SrtA_△N40(D56E)_, as indicated by the *k*_*cat*_*/K*_*m*_ ratios. The most apparent effect on SrtA catalysis was produced by the mutation D56E. The *k*_*cat*_*/K*_*m*_ values of SrtA_△N40(UA159)_ and SrtA_△N40(R157H)_ were approximately 4.1 and 3.0 times the *k*_*cat*_*/K*_*m*_ of SrtA_△N40(D56E)_, respectively. The *k*_*cat*_*/K*_*m*_ of SrtA_△N40(UA159)_ was slightly higher than the *k*_*cat*_*/K*_*m*_ of SrtA_△N40(R157H)._

## Discussion

*S. mutans* is the primary pathogen of dental caries, and because of a variety of different genetic events, *S. mutans* strains exhibit considerable phenotypic variation and differ in cariogenicity [13]. The protease SrtA is an important virulence factor that catalyses the cell wall anchoring of surface proteins containing an LPXTG motif [7] and the polymorphisms of the *srtA* gene could lead to variations in cariogenic capacity [1].

Previously, we performed two epidemiological investigations to explore and compare the genetic polymorphisms of the *srtA* gene among clinical strains of *S. mutans* that were isolated from children with distinct caries status [15, 16]. The results indicated that almost all clinical isolates harboured point mutations when *S. mutans* UA159 served as the template strain. Although the locations and periods of the two epidemiological investigations differed, the two epidemiological investigations yielded similar results. The T168G mutation was primarily observed in the caries-free group, whereas the G470A mutation was mainly detected in the caries-active group [16]. Based on these results, this study was conducted to assess the effects of missense mutations T168G and G470A in the *srtA* gene on the activity of the SrtA enzyme, which has not been previously reported.

A single point mutation in *srtA* gene was demonstrated to be able to completely change the enzyme activity. The *srtA* genes in *S. mutans* Ingbritt and *S. mutans* NG5 both contain nonsense mutations that cause premature termination and result in the production of incomplete SrtA enzymes and defective cell wall sorting activity [14, 25]. The *srtA* gene of *S. mutans* NG5 contains a stop codon arising from a single base substitution from G to T at a GAA codon that is 70 amino acids upstream of the putative active site of the enzyme [14]. The generation of a new termination codon in the *srtA* gene of *S. mutans* Ingbritt arises from a deletion of 11 bp [25]. In *Staphylococcus aureus*, mutations at H120, C184 and R197, the catalytic triad of SrtA, could affect the enzymatic activity. For example, the point mutated sortases SrtA_△N24(H120Q)_, SrtA_△N24(C184S)_ and SrtA_△N24(R197A)_ were expressed as the recombinant protein that coded by *srtA*_*△N72(C360A)*_, *srtA*_*△N72(T550A)*_, and *srtA*_*△N72(C589G/G590C)*_, respectively. Compared with the wild-type SrtA, the enzymatic activities of these point mutated sortases decreased dramatically [26]. However, the mutation that is not at the active site of SrtA could also influence the enzymatic activity. For instance, the average activity of point mutated sortase SrtA_△N59(I123G)_, which was expressed as the recombinant protein that coded by *srtA*_*△N177(A367G/T368G)*_, was also lower than the average activity of wild-type SrtA [23].

Our study showed that missense mutations arising from single base substitutions of T168G and G470A in the *srtA* gene in *S. mutans* could also result in changes in enzyme activity. Compared with the amino acid sequence of SrtA_△N40(UA159)_, the mutant enzymes SrtA_△N40(D56E)_ and SrtA_△N40(R157H)_ contained single amino acid substitutions from D to G at the 56th amino acid residue and from R to H at the 157th amino acid residue, respectively. Although the D56E and R157H mutations in the amino acid sequence did not lead to marked changes in the affinity of SrtA for the Dabcyl-QALPNTGEE-Edans substrate, the catalytic efficiency of SrtA_△N40(D56E)_ was decreased compared with those of SrtA_△N40(UA159)_ and SrtA_△N40(R157H)_. The D56E mutation significantly affected SrtA catalysis. And the enzymatic activity of SrtA_(D56E)_, which is primarily expressed in the caries-free group, was notably decreased compared with the enzymatic activity of SrtA_△N40(UA159)_ and SrtA_△N40(R157H)_, which is primarily expressed in the caries-active group (Table 1). This difference may be responsible for the significantly lower incidence of caries in the caries-free group than in the caries-active group. Nevertheless, dental caries is an infective bacterial disease characterized by a multi-factorial pathology, and many factors other than the *srtA* gene of *S. mutans* contribute to dental caries.

The mutation data may be explained through correlation with the physical-chemical characteristics of amino acids [27]; the physical-chemical characteristics such as charge and size more or less differ among D, E, R and H. However, the mechanisms by which amino acid mutations affect protein function are complex and related to many factors [23, 27–30], including protein dimerization and structure, among others. Additionally, some cases could not be easily explained or identified by structure alone. Multiple amino acid sequence alignments of sortase enzymes with determined structures in closely related species indicated that the catalytic triad of SrtA in *S. mutans* was composed of H139, C205 and R213 [22]. Thus, amino acid residues D56 and R157 do not belong to the catalytic triad, which is important for enzyme activity. To preliminarily explore the possible mechanism of the effects of the D56E and R157H amino acid mutations on SrtA enzyme activities, we analysed the generated SrtA_△N40(UA159)_ and mutants for dimerization using native PAGE. The results showed that the recombinant SrtA enzymes existed primarily in both dimeric and monomeric forms, which was consistent with previous study [23]. No apparent differences in the ratios of dimeric/monomeric SrtA_△N40(UA159)_, SrtA_△N40(D56E)_ and SrtA_△N40(R157H)_ were found, indicating that SrtA_△N40(D56E)_ and SrtA_△N40(R157H)_ were similar to SrtA_△N40(UA159)_ in terms of monomer-dimer equilibrium and that the effects of the D56E and R157H mutations on the enzyme activities may not be related to SrtA dimerization. Further in-depth studies are needed to investigate the exact mechanism by which the D56E and R157H amino acid mutations affect the enzyme activities.

This study was subject to certain limitations. Research limitations precluded us from investigating the effects of D56E and R157H mutations on the structure or conformation of SrtA. Mutant strains of *S. mutans* should be constructed in future studies to observe the effects of the D56E and R157H mutations in SrtA on strain phenotypes, such as adhesion and biofilm formation. However, the selection of mutation sites and mutation types in our study were based on two previous clinical epidemiological investigations whereas traditional studies of random mutations and specific studies of enzyme active sites or domains did not rely on such epidemiological investigations [15, 16]. Therefore, the results of this study were an approximation of the clinical condition and manifestation of caries, which is significant for the guidance of clinical preventive services. To the best of our knowledge, the effects of the D56E and R157H mutations on the catalytic activity of SrtA have not yet been studied in the context of missense mutations of *srtA* in *S. mutans*.

## Conclusions

The reasons for the differences in cariogenicity among clinical isolates of *S. mutans* are complex. The results of the present study suggest that the diversity of the *srtA* gene can lead to the differences in enzyme activity in clinical isolates of *S. mutans*. Specifically, the T168G mutation in the *srtA* gene of *S. mutans* can decrease the enzyme activity.

### Availability of data and materials

The datasets supporting the conclusions of this article are included within the article and its Additional file 1.

## Additional file

Additional file 1:
**Table S1.** Data of the enzyme activity assay of SrtA_△N40(UA159)_. **Table S2.** Data of the enzyme activity assay of SrtA_△N40(D56E)_. **Table S3.** Data of the enzyme activity assay of SrtA_△N40(R157H)_. (DOCX 39 kb)

## Abbreviations

Dabcyl-QALPNTGEE-Edans: 4-(4-dimethylamino phenylazo)benzoic acid (Dabcyl)-QALPETGEE -5-[(2-aminoethyl)amino]naphthalene-1-sulphonic acid (Edans)
DNA: deoxyribonucleic acid
*E. coli*: *Escherichia coli*
GbpC: glucan binding protein C
IPTG: isopropyl β-D-1-thiogalactopyranoside
*k*_*cat*_: catalytic rate constant
*k*_*cat*_*/K*_*m*_: catalytic efficiency
*K*_*m*_: michaelis constant
native PAGE: non-denaturing polyacrylamide gel electrophoresis
PCR: polymerase chain reaction
*S. mutans*: *Streptococcus mutans*
SDS-PAGE: sodium dodecyl sulphate–polyacrylamide gel electrophoresis
SrtA: sortase A
*Vmax*: maximum velocity
WapA: wall-associated protein A

## References

[1] Werneck RI, Mira MT, Trevilatto PC (2010). A critical review: an overview of genetic influence on dental caries. Oral Dis.

[2] Krzyściak W, Jurczak A, Kościelniak D, Bystrowska B, Skalniak A (2014). The virulence of Streptococcus mutans and the ability to form biofilms. Eur J Clin Microbiol Infect Dis.

[3] Sato Y, Okamoto-Shibayama K, Azuma T. A mechanism for extremely weak SpaP-expression in Streptococcus mutans strain Z1. J Oral Microbiol. 2011;3. doi:10.3402/jom.v3i0.5495. 10.3402/jom.v3i0.5495 PMC3086597 21541094

[4] Tamura H, Yamada A, Kato H (2014). Molecular characterization of the dextran-binding lectin B gene dblB of streptococcus criceti in Streptococcus mutans strain GS-5 with mutations in both gbpC and spaP genes. Genes Genet Syst.

[5] Li Y, Liu Z, Zhang Y, Su QP, Xue B, Shao S, et al. Live-cell and super-resolution imaging reveal that the distribution of wall-associated protein A is correlated with the cell chain integrity of Streptococcus mutans. Mol Oral Microbiol. 2015; [Epub ahead of print]. doi:10.1111/omi.12100. 10.1111/omi.12100 25891147

[6] Otsuka R, Imai S, Murata T, Nomura Y, Okamoto M, Tsumori H (2015). Application of chimeric glucanase comprising mutanase and dextranase for prevention of dental biofilm formation. Microbiol Immunol.

[7] Marraffini LA, Dedent AC, Schneewind O (2006). Sortases and the art of anchoring proteins to the envelopes of Gram-positive bacteria. Microbiol Mol Biol Rev.

[8] Nobbs AH, Lamont RJ, Jenkinson HF (2009). Streptococcus adherence and colonization. Microbiol Mol Biol Rev.

[9] Spirig T, Weiner EM, Clubb RT (2011). Sortase enzymes in Gram-positive bacteria. Mol Microbiol.

[10] Yamaguchi M, Terao Y, Ogawa T, Takahashi T, Hamada S, Kawabata S (2006). Role of streptococcus sanguinis sortase A in bacterial colonization. Microbes Infect.

[11] Lee SF, Boran TL (2003). Roles of sortase in surface expression of the major protein adhesin P1, saliva-induced aggregation and adherence, and cariogenicity of Streptococcus mutans. Infect Immun.

[12] Lévesque CM, Voronejskaia E, Huang YC, Mair RW, Ellen RP, Cvitkovitch DG (2005). Involvement of sortase anchoring of cell wall proteins in biofilm formation by Streptococcus mutans. Infect Immun.

[13] Lembo FL, Longo PL, Ota-Tsuzuki C, Rodrigues CR, Mayer MP (2007). Genotypic and phenotypic analysis of Streptococcus mutans from different oral cavity sites of caries-free and caries-active children. Oral Microbiol Immunol.

[14] Lee SF, McGavin MK (2004). Identification of a point mutation resulting in loss of cell wall anchoring activity of SrtA of Streptococcus mutans NG5. Infect Immun.

[15] Zhang XH, Zhou Y, Zhi QH, Tao Y, Lin HC (2012). Genetic polymorphisms of the sortase A gene and early childhood caries in two-year-old children. Arch Oral Biol.

[16] Yu LX, Tao Y, Qiu RM, Zhou Y, Zhi QH, Lin HC (2015). Genetic polymorphisms of the sortase A gene and social-behavioural factors associated with caries in children: a case-control study. BMC Oral Health.

[17] Lu C, Zhu J, Wang Y, Umeda A, Cowmeadow RB, Lai E (2007). Staphylococcus aureus sortase A exists as a dimeric protein *in vitro*. Biochemistry.

[18] Ilangovan U, Ton-That H, Iwahara J, Schneewind O, Clubb RT (2001). Structure of sortase, the transpeptidase that anchors proteins to the cell wall of Staphylococcus aureus. Proc Natl Acad Sci U S A.

[19] Deng FK, Zhang L, Wang YT, Schneewind O, Kent SB (2014). Total chemical synthesis of the enzyme sortase A(ΔN59) with full catalytic activity. Angew Chem Int Ed Engl.

[20] Hu P, Huang P, Chen WM (2013). Curcumin inhibits the Sortase A activity of the Streptococcus mutans UA159. Appl Biochem Biotechnol.

[21] Huang P, Hu P, Zhou SY, Li Q, Chen WM (2014). Morin inhibits sortase A and subsequent biofilm formation in Streptococcus mutans. Curr Microbiol.

[22] Wallock-Richards DJ, Marles-Wright J, Clarke DJ (2015). Molecular basis of Streptococcus mutans sortase A inhibition by the flavonoid natural product trans-chalcone. Chem Commun (Camb).

[23] Zhu J, Lu C, Standland M, Lai E, Moreno GN, Umeda A (2008). Single mutation on the surface of Staphylococcus aureus Sortase A can disrupt its dimerization. Biochemistry.

[24] Swamy M, Siegers GM, Minguet S (2006). Blue native polyacrylamide gel electrophoresis (BN-PAGE) for the identification and analysis of multiprotein complexes. Sci STKE.

[25] Igarashi T (2004). Deletion in sortase gene of Streptococcus mutans Ingbritt. Oral Microbiol Immunol.

[26] Frankel BA, Tong Y, Bentley ML, Fitzgerald MC, McCafferty DG (2007). Mutational analysis of active site residues in the Staphylococcus aureus transpeptidase SrtA. Biochemistry.

[27] Taylor WR (1986). The classification of amino acid conservation. J Theor Biol.

[28] Studer R, Dessailly B, Orengo C (2013). Residue mutations and their impact on protein structure and function: detecting beneficial and pathogenic changes. Biochem J.

[29] Teng S, Srivastava AK, Schwartz CE, Alexov E, Wang L (2010). Structural assessment of the effects of amino acid substitutions on protein stability and protein-protein interaction. Int J Comput Biol Drug Des.

[30] Yates CM, Sternberg MJ (2013). The effects of non-synonymous single nucleotide polymorphisms (nsSNPs) on protein-protein interactions. J Mol Biol.

