# Supplementary material for: Effects of missense mutations in sortase A gene on enzyme activity in Streptococcus mutans
Source: BMC Oral Health. 2016 Apr 11;16:47. doi: 10.1186/s12903-016-0204-1 (PMC4827206; doi:10.1186/s12903-016-0204-1)
Supplement: Additional file 1: — Table S1. Data of the enzyme activity assay of SrtA△N40(UA159). Table S2. Data of the enzyme activity assay of SrtA△N40(D56E). Table S3. Data of the enzyme activity assay of SrtA△N40(R157H). (DOCX 39 kb) [file 12903_2016_204_MOESM1_ESM.docx]

Additional file 1

The supporting data regarding the enzyme activities of SrtA_△N40(UA159)_ and the mutant enzymes are provided in Tables S1, S2 and S3. All reported values in Table 1 in the article are the means of triplicate assays.

**Table S1 Data of the enzyme activity assay of SrtA_△N40(UA159)_**

| Assay | Time | *[E]* | *[S]* | *v* | *Vmax* | *K_m_* | *k_cat_* | *k_cat_/K_m_* |
| --- | --- | --- | --- | --- | --- | --- | --- | --- |
| No. |  |  |  | (×10^-3^) | (×10^-3^) |  | (×10^-3^) | (×10^-4^) |
|  | (min) | (μM) | (μM) | (μM·s^-1^) | (μM·s^-1^) | (μM) | (s^-1^) | (μM^-1^·s^-1^) |
| 1 | 30 | 1.2 | 0.2 | 0.05 | 3.71 | 15.21 | 3.01 | 1.98 |
|  | 30 | 1.2 | 0.4 | 0.10 |  |  |  |  |
|  | 30 | 1.2 | 0.8 | 0.18 |  |  |  |  |
|  | 30 | 1.2 | 1.6 | 0.34 |  |  |  |  |
|  | 30 | 1.2 | 3.2 | 0.61 |  |  |  |  |
|  | 30 | 1.2 | 6.4 | 1.20 |  |  |  |  |
|  | 30 | 1.2 | 12.8 | 1.90 |  |  |  |  |
|  |  |  |  |  |  |  |  |  |
| 2 | 30 | 1.2 | 0.2 | 0.05 | 3.92 | 15.61 | 3.17 | 2.03 |
|  | 30 | 1.2 | 0.4 | 0.10 |  |  |  |  |
|  | 30 | 1.2 | 0.8 | 0.19 |  |  |  |  |
|  | 30 | 1.2 | 1.6 | 0.35 |  |  |  |  |
|  | 30 | 1.2 | 3.2 | 0.63 |  |  |  |  |
|  | 30 | 1.2 | 6.4 | 1.24 |  |  |  |  |
|  | 30 | 1.2 | 12.8 | 1.96 |  |  |  |  |
|  |  |  |  |  |  |  |  |  |
| 3 | 30 | 1.2 | 0.2 | 0.05 | 3.63 | 15.68 | 2.94 | 1.87 |
|  | 30 | 1.2 | 0.4 | 0.09 |  |  |  |  |
|  | 30 | 1.2 | 0.8 | 0.17 |  |  |  |  |
|  | 30 | 1.2 | 1.6 | 0.32 |  |  |  |  |
|  | 30 | 1.2 | 3.2 | 0.58 |  |  |  |  |
|  | 30 | 1.2 | 6.4 | 1.14 |  |  |  |  |
|  | 30 | 1.2 | 12.8 | 1.80 |  |  |  |  |

*[E]*: enzyme concentration; *[S]*: substrate concentration; *v*: velocity; *Vmax*: maximum velocity; *K_m_*: Michaelis constant; *k_cat_*: catalytic rate constant; *k_cat_/K_m_*: catalytic efficiency.

**Table S2 Data of the enzyme activity assay of SrtA_△N40(D56E)_**

| Assay | Time | *[E]* | *[S]* | *v* | *Vmax* | *K_m_* | *k_cat_* | *k_cat_/K_m_* |
| --- | --- | --- | --- | --- | --- | --- | --- | --- |
| No. |  |  |  | (×10^-3^) | (×10^-3^) |  | (×10^-3^) | (×10^-4^) |
|  | (min) | (μM) | (μM) | (μM·s^-1^) | (μM·s^-1^) | (μM) | (s^-1^) | (μM^-1^·s^-1^) |
| 1 | 30 | 1.2 | 0.2 | 0.01 | 1.03 | 15.66 | 0.84 | 0.53 |
|  | 30 | 1.2 | 0.4 | 0.03 |  |  |  |  |
|  | 30 | 1.2 | 0.8 | 0.05 |  |  |  |  |
|  | 30 | 1.2 | 1.6 | 0.09 |  |  |  |  |
|  | 30 | 1.2 | 3.2 | 0.17 |  |  |  |  |
|  | 30 | 1.2 | 6.4 | 0.33 |  |  |  |  |
|  | 30 | 1.2 | 12.8 | 0.51 |  |  |  |  |
|  |  |  |  |  |  |  |  |  |
| 2 | 30 | 1.2 | 0.2 | 0.01 | 0.93 | 15.03 | 0.76 | 0.50 |
|  | 30 | 1.2 | 0.4 | 0.02 |  |  |  |  |
|  | 30 | 1.2 | 0.8 | 0.05 |  |  |  |  |
|  | 30 | 1.2 | 1.6 | 0.09 |  |  |  |  |
|  | 30 | 1.2 | 3.2 | 0.16 |  |  |  |  |
|  | 30 | 1.2 | 6.4 | 0.31 |  |  |  |  |
|  | 30 | 1.2 | 12.8 | 0.48 |  |  |  |  |
|  |  |  |  |  |  |  |  |  |
| 3 | 30 | 1.2 | 0.2 | 0.01 | 0.77 | 15.34 | 0.63 | 0.41 |
|  | 30 | 1.2 | 0.4 | 0.02 |  |  |  |  |
|  | 30 | 1.2 | 0.8 | 0.04 |  |  |  |  |
|  | 30 | 1.2 | 1.6 | 0.07 |  |  |  |  |
|  | 30 | 1.2 | 3.2 | 0.13 |  |  |  |  |
|  | 30 | 1.2 | 6.4 | 0.25 |  |  |  |  |
|  | 30 | 1.2 | 12.8 | 0.39 |  |  |  |  |

*[E]*: enzyme concentration; *[S]*: substrate concentration; *v*: velocity; *Vmax*: maximum velocity; *K_m_*: Michaelis constant; *k_cat_*: catalytic rate constant; *k_cat_/K_m_*: catalytic efficiency.

**Table S3 Data of the enzyme activity assay of SrtA_△N40(R157H)_**

| Assay | Time | *[E]* | *[S]* | *v* | *Vmax* | *K_m_* | *k_cat_* | *k_cat_/K_m_* |
| --- | --- | --- | --- | --- | --- | --- | --- | --- |
| No. |  |  |  | (×10^-3^) | (×10^-3^) |  | (×10^-3^) | (×10^-4^) |
|  | (min) | (μM) | (μM) | (μM·s^-1^) | (μM·s^-1^) | (μM) | (s^-1^) | (μM^-1^·s^-1^) |
| 1 | 30 | 1.2 | 0.2 | 0.04 | 3.09 | 15.36 | 2.50 | 1.63 |
|  | 30 | 1.2 | 0.4 | 0.08 |  |  |  |  |
|  | 30 | 1.2 | 0.8 | 0.15 |  |  |  |  |
|  | 30 | 1.2 | 1.6 | 0.28 |  |  |  |  |
|  | 30 | 1.2 | 3.2 | 0.50 |  |  |  |  |
|  | 30 | 1.2 | 6.4 | 0.99 |  |  |  |  |
|  | 30 | 1.2 | 12.8 | 1.56 |  |  |  |  |
|  |  |  |  |  |  |  |  |  |
| 2 | 30 | 1.2 | 0.2 | 0.03 | 2.59 | 15.42 | 2.09 | 1.36 |
|  | 30 | 1.2 | 0.4 | 0.07 |  |  |  |  |
|  | 30 | 1.2 | 0.8 | 0.13 |  |  |  |  |
|  | 30 | 1.2 | 1.6 | 0.23 |  |  |  |  |
|  | 30 | 1.2 | 3.2 | 0.42 |  |  |  |  |
|  | 30 | 1.2 | 6.4 | 0.83 |  |  |  |  |
|  | 30 | 1.2 | 12.8 | 1.31 |  |  |  |  |
|  |  |  |  |  |  |  |  |  |
| 3 | 30 | 1.2 | 0.2 | 0.03 | 2.68 | 15.28 | 2.17 | 1.42 |
|  | 30 | 1.2 | 0.4 | 0.07 |  |  |  |  |
|  | 30 | 1.2 | 0.8 | 0.13 |  |  |  |  |
|  | 30 | 1.2 | 1.6 | 0.24 |  |  |  |  |
|  | 30 | 1.2 | 3.2 | 0.44 |  |  |  |  |
|  | 30 | 1.2 | 6.4 | 0.86 |  |  |  |  |
|  | 30 | 1.2 | 12.8 | 1.36 |  |  |  |  |

*[E]*: enzyme concentration; *[S]*: substrate concentration; *v*: velocity; *Vmax*: maximum velocity; *K_m_*: Michaelis constant; *k_cat_*: catalytic rate constant; *k_cat_/K_m_*: catalytic efficiency.
